# Imatinib independent aberrant methylation of NOV/CCN3 in chronic myelogenous leukemia patients: a mechanism upstream of BCR-ABL1 function?

**DOI:** 10.1186/s12964-019-0350-6

**Published:** 2019-04-23

**Authors:** Mousa Vatanmakanian, Mahmood Tavallaie, Shirin Ghadami

**Affiliations:** Human Genetics Research Center, Baqiyatallah University of Medical Sciences, Mollasadra Ave., Vanak Square, Tehran, Iran

**Keywords:** NOV/CCN3, DNA methylation, Chronic myelogenous leukemia (CML), BCR-ABL1

## Abstract

**Background:**

The NOV gene product, CCN3, has been reported in a diverse range of tumors to serve as a negative growth regulator, while acting as a tumor suppressor in Chronic Myelogenous Leukemia (CML). However, the precise mechanism of its silencing in CML is poorly understood. In the current study, we aimed to query if the gene regulation of CCN3 is mediated by the promoter methylation in the patients with CML. In addition, to clarify whether the epigenetic silencing is affected by BCR-ABL1 inhibition, we assessed the methylation status in the patients at different time intervals following the tyrosine kinase inhibition using imatinib therapy, as the first-line treatment for this type of leukemia.

**Methods:**

To address this issue, we applied bisulfite-sequencing technique as a high-resolution method to study the regulatory segment of the CCN3 gene. The results were analyzed in newly diagnosed CML patients as well as following imatinib therapy. We also evaluated the correlation of CCN3 promoter methylation with BCR-ABL1 levels.

**Results:**

Our findings revealed that the methylation occurs frequently in the promoter region of CML patients showing a significant increase of the methylated percentage at the CpG sites compared to normal individuals. Interestingly, this hypermethylation was indicated to be independent of BCR-ABL1 titers in both groups, which might suggest a mechanism beyond the BCR-ABL1 function.

**Conclusion:**

Despite suggesting that the CCN3 hypermethylation acts as a molecular mechanism independent of BCR-ABL1 function in CML patients, this scenario requires further validation by complementary experiments. In the case of acting upstream of BCR-ABL1 signaling, the methylation marker can provide early detection and a novel platform for targeted epigenetic modifiers for efficient treatment in imatinib resistant patients.

## Background

Chronic Myelogenous Leukemia (CML) is categorized as a myeloproliferative disorder which constitutes approximately 25% of leukemia cases. The disease was originally discovered to be resulted from a clonal expansion of an affected hematopoietic stem cell [1–3]. The well-known triggering molecular event initiating the CML is a chromosomal translocation between chromosomes 9 and 22, t (9;22), leading to the formation of a fusion onco-protein (BCR-ABL1) which is characterized by an overactive tyrosine kinase activity [4]. Much like other enzymes in this class, the ABL1 gene product is assumed to play roles in controlling cellular growth and response to DNA damage [5, 6]. Although the precise underlying mechanism through which this alteration results in leukemogenesis is not well clarified, the ultimate event is the proliferation of the stem cell pool with uncontrolled expansion of mature cells from myeloid lineage. The association of BCR-ABL1 expression has been established with a wide range of pathways involved in cell proliferation, survival, and apoptosis resistance [7]. The upstream key events, however, by which the BCR-ABL1 positive stem cells dominate the bone marrow leading to CML formation is still under query. Currently, the CML cases are clinically diagnosed in chronic phase, and the onset of disease remains elusive days or years prior to detection.

Over the last two decades, much progress has been made to reveal phenotypic outcomes associated with post-translational modifications in histone, DNA modification, and non-coding RNAs as integral parts of epigenetic machinery. Notably, large cohort of studies has reported the deregulated DNA methylation in multiple genes which associates with disease progression toward malignant behaviors and more advanced clinical staging [8, 9]. It is hypothesized that if detectable epigenetic signatures in the human genome functioning beyond the traditional culprits of cancer initiators can be revealed, they might serve as the candidate markers for disease prediction or even the prophylactic intervention using targeted epigenetic modifiers.

With respect to newer technologies, we have used bisulfite sequencing method to elucidate the DNA methylation profile of the NOV/CCN3 gene which is implicated in the clonal evolution of CML cancer stem cells. This study was aimed to evaluate whether the methylation alteration of the stem cell-associated gene, Nephroblastoma Overexpressed (NOV), occurs beyond the function of BCR-ABL fusion gene.

It has been established that Cellular Communication Network Factor 3 (CCN3) protein plays an immediate-early roles in key cellular events such as proliferation, cellular adhesion, migration, cell fate determination, and survival [10–13]. Its crucial role in modulating the functional integrity of hematopoietic stem and progenitor cells has been also well-evidenced [14]. Although acting as metastatic accelerator in many solid tumors including Ewings Sarcoma [15], osteosarcoma [16], prostate neoplasia [17], breast [18], and bladder cancer [19]; the NOV gene was shown to be silenced in CML serving as a tumor suppressor and growth regulator [20–22]. Nevertheless, the underlying mechanism by which the gene is down-regulated is partially elucidated [20, 22].

Enrichment of DNA methylation in the CpG segments is a well-known mechanism influencing gene promoter and regulatory regions. This epigenetic-based gene silencing mostly contributes to inactivate the tumor suppressor genes in malignant cells [23, 24]. Owing to the previous data on the contribution of CCN3 during the HSC maintenance as well as the lower expression of the gene in CML, we were wondering whether the NOV gene is controlled via promoter methylation. Our study was designed also to address if the possible changes in DNA methylation of NOV gene remain intact following the imatinib treatment as a BCR-ABL1 suppressor. Although the evidence provided by our study might not be adequate to clearly estimate the hierarchical events during BCR-ABL1-positive HSCs domination, we revealed that hypermethylation that occurs in CML patients is not influenced by targeting the BCR-ABL1 tyrosine kinase.

## Methods

### Patients and sample collection

The peripheral blood samples were collected in tubes containing K2-EDTA as anti-coagulant from 20 newly diagnosed CML patients between October 2017 and July 2018 (median age of 49 years, ranging from 21 to 75 years; male: 9; female: 11) in the Gholhak Laboratory, Tehran, Iran approved by the institutional standard protocols. The diagnosis was performed based on morphology, chromosome analysis using karyotyping which was definitely confirmed by the measurement of BCR-ABL1 fusion gene titers using absolute quantification using real-time PCR. Nine patients were classified in chronic phase, 5 in accelerated phase and 6 in blastic phase. A written consent was provided for each individual participant and the study was approved by the Medical Ethics Committee of Baqiyatallah University of Medical Sciences.

### Molecular and hematologic response evaluation

The subjects were then followed up for 11 months following treatment and the second sample was collected from the participants at various time intervals of imatinib therapy (6–11 months). Twenty five samples were also taken from healthy individuals as a control group and confirmed to have normal CBC and negative for BCR-ABL1 fusion gene transcripts. The imatinib mesylate dosages were administered for the patients based on hematologic and non-hematologic toxicities, ranging from 200 to 400 mg P.O. daily for chronic phase, 300–800 for accelerated phase, and 600–800 for blastic phase. The Complete Hematologic Responses (CHR) were evaluated as defined by the WBC count bellow 10 × 10^9^/L, the absence of immature cells (including blast cells, myelocytes, and promyelocytes) using microscopic assessment of peripheral blood, and undetectible signs of leukemia such as palpable splenomegaly lasting for at least 4 weeks. In addition, an undetectable BCR-ABL/ABL transcripts was defined as a Complete Molecular Response (CMR), while 3-log or more decline in titers compared to the baseline levels defines a Major Molecular Response (MMR). No Molecular Response (NMR) was also defined as detecting increasing changes in BCR-ABL/ABL compared with baseline; and a Partial Molecular Response (PMR) was considered as a reduction in BCR-ABL/ABL transcript less than 3-log.

### DNA extraction and bisulfite conversion

The genomic DNA samples were isolated from the peripheral blood using the KBC Blood DNA Extraction Kit with the standard salting out/proteinase K method (Kosar Biotechnology Co. Tehran, Iran). The samples were then quantified using a Nano-drop spectrophotometer (Thermofisher Scientific) and ensured to have a high concentration and purity, which is optimal for efficient bisulfite conversion (the concentration of more than 200 ng/μl, OD260/230 of 2.0–2.2; and 260/280 of 1.8–2.0). The DNA samples were then treated with sodium bisulfite according to the manufacturer’s instructions (EpiTect Bisulfite Kit, QIAGEN, Hilden, Germany). The bisulfite treatment catalyzes the deamination of all the unmethylated cytosine (uC) nucleotides to uracil (U) or thymidine (T) nucleotides and leaves the methylated cytosine (mC) unchanged. For optimal results, we input the amount of starting DNA from 200 to 500 ng in the bisulfite modification process.

### Primer design and bisulfite sequencing PCR (BSP)

The MethPrimer tool (http://www.urogene.org/cgi-bin/methprimer/methprimer.cgi) was used to predict the CpG Island based on the promoter sequence provided from UCSC database, and BSP primers were designed according to the standard parameters and synthesized by GenFanAvaran Co. (Tehran, Iran). The PCR reactions were performed using 12 μl of Taq DNA Polymerase 2x Master Mix RED with 2 mM MgCl2 final concentration (Ampliqon, Odense M, Denmark); 2 μl bisulfite treated DNA (approximately 50 ng), 0.5 μl of each forward and reverse primers (10pM), and ddH2O to the final volume of 25 μl. The reactions were conducted in a SimpliAmp thermal cycler device (Applied Biosystems) with a primary denaturation for 5 min at 95 °C followed by 40 cycles of denaturation at 95 °C for 30 Sec, annealing temperature with descending touchdown system (− 0.2 °C) starting at 59 °C for 45 Sec, an extension at 72 °C for 45 Sec; and a final extension at 72 °C for 10 min. Following DNA cleanup procedure using the shrimp alkaline phosphatase enzyme for PCR product purification (Thermofisher Scientific). The products were then sequenced with an ABI PRISM BigDye terminator sequencing kit v1.1 (Life Technologies), and directly analyzed by an automated ABI 3130 Genetic Analyzer (Life Technologies).

### Data interpretation and statistical analysis

Sequence alignment, methylation analysis, and interpretation were performed using the BiQ Analyzer software v2.02 [25]. The statistical analysis and lollipop-style representation of methylation data were provided using the QUMA online tool ((http://quma.cdb.riken.jp/). Accordingly, as the methylation status does not distribute normally, particularly in case of hypo- and hyper-methylation, the significance of difference between two sets of entire CpG sites in BSP groups is determined by the Mann-Whitney U-test which is a non-parametric test for evaluation of samples with such distributions. Also, the difference between two BSP groups at each CpG site is measured by the Fisher’s exact test as non-parametric statistical significance test to assess whether nonrandom correlation exists between two categorical data. The *p*-value showing the independence of CpG methylation between two CpG sites was calculated from Fisher’s exact test and the results of less than 0.05 were considered statistically significant. The statistical analysis of numeric data to determine other correlations were implemented using the SPSS 20.0 software package (SPSS, Chicago, IL, USA).

## Results

**Fig. 1:**
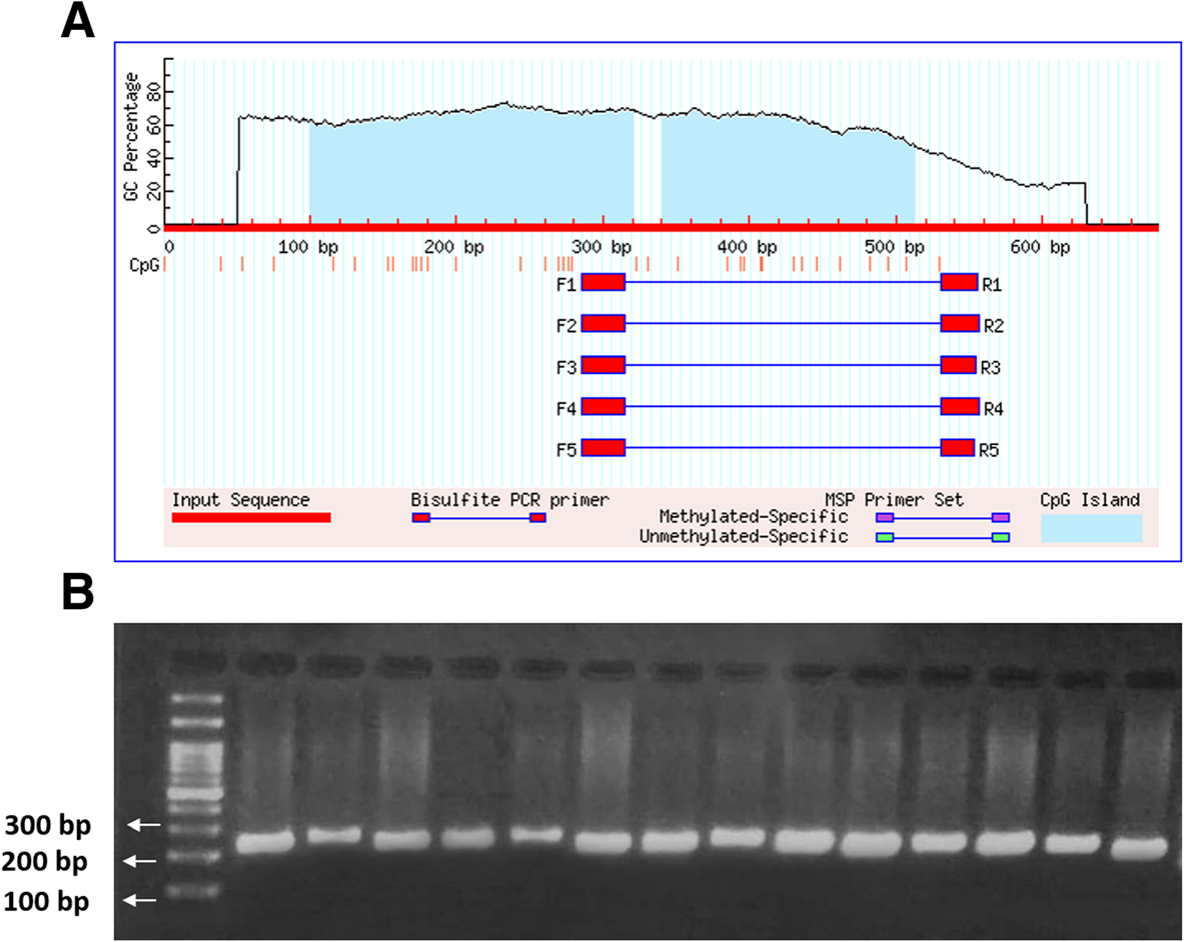
Schematic diagram of the analyzed BSP primer. **a**: The schematic representation of the predicted CpG islands (blue region), CpG sites (small red bars), and Bisulfite PCR primer positions (F1: Forward primer; R1: Reverse primer). **b**: The PCR bands of the samples following the NOV amplification showing the product size of 271 bp

Two CpG islands were predicted using the MethPrimer tool in proximity of the transcriptional start site of the NOV gene. A schematic representation of the predicted islands and the position of the primers are depicted in Fig. 1a. The island 1 was positioned at nt − 200 to + 20 and Island 2 at nt + 41 to + 131 related to the first exon. The primer was designed to create an amplicon spanning all the exon 1 and a part of promoter (both prone to be regulated by DNA methylation). CpG rich region with 16 CpG sites from nt − 16 bp to + 255 bp resulting in a product with 271 bp was considered as the region for designing BSP specific primers, NOV-F1: 5′ AAGGAGAGTAGTATTTATAGTTAATTGTTA-3′, NOV-R1: 5′- ATACACCAAAATAATACAAAATCAC-3′. The target PCR sequenced region and the CpG positions are shown below:

- **aaggagagcagcacccacagccaattgcca**tggcaaccc**CG**gggtt**CG**ttccacttccccacccagc**CG**atctcccccctcctccctgcactgcagccaac**CG**gcttgtg**CGCG**tcccaggag**CGCG**ctataaaacctgtgctggg**CG**tgat**CG**gcaagcac**CG**gaccagggggaagg**CG**agcagtgccaatctacag**CG**aagaaagtct**CG**tttggtaaaag**CG**agaggggaaagcctgagctg**CGtgactctgtattactttggtgcat.**

### The CML patinets show a hypermethylated NOV gene compared to normal individuals

**Fig. 2:**
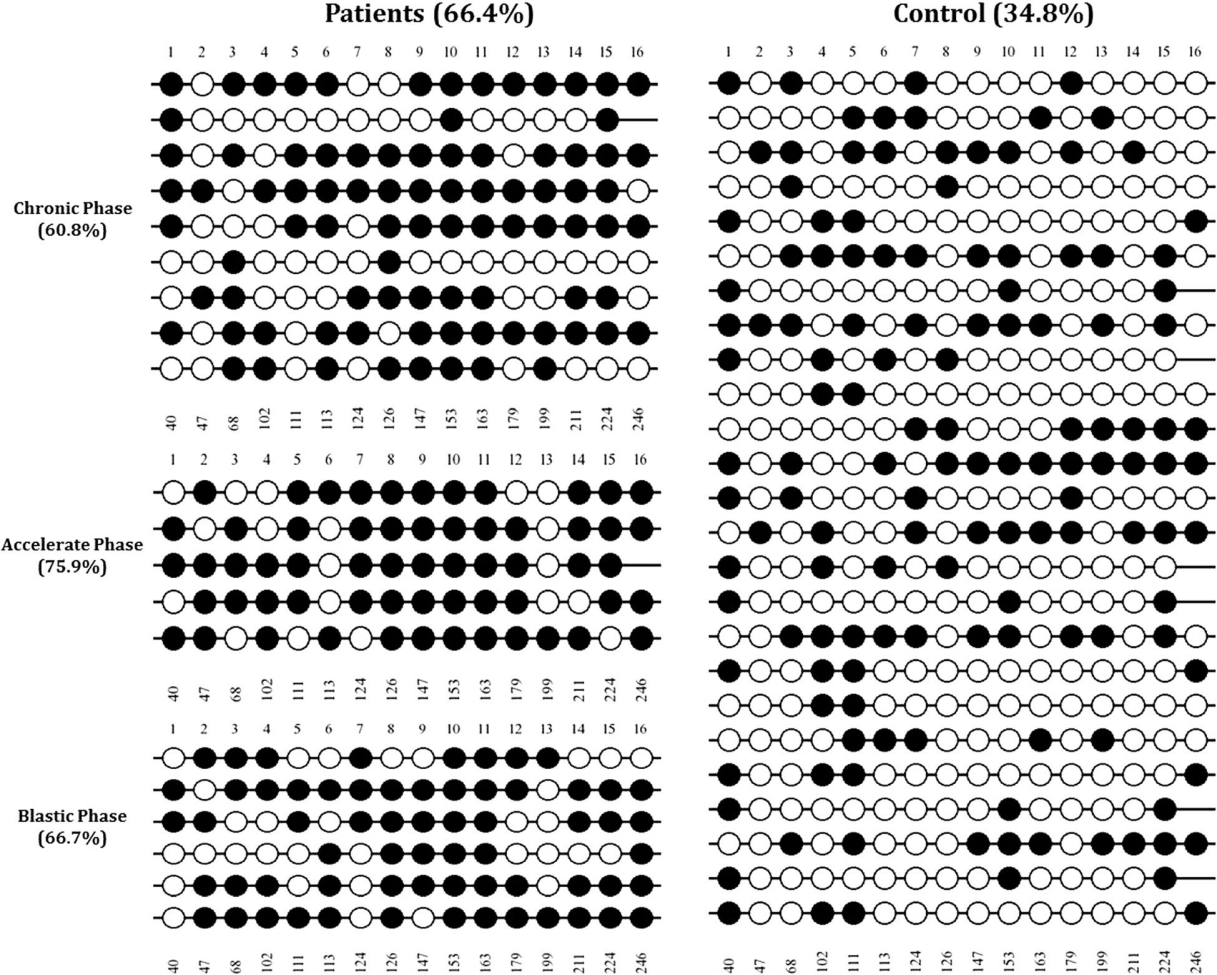
The lollipops representation of the methylation data. All the CML patients in various clinical staging showed greater levels of CpG methylation (left panel) compared to normal samples (right sample). The number of CpG position is inserted at the top of each position and the nucleotide position in the target sequence is depicted at the bottom of each CpG site. The Black circles represent the methylated sites while the white circles indicate the unmethylated positions. The diagram was created using QUMA online software

**Fig. 3:**
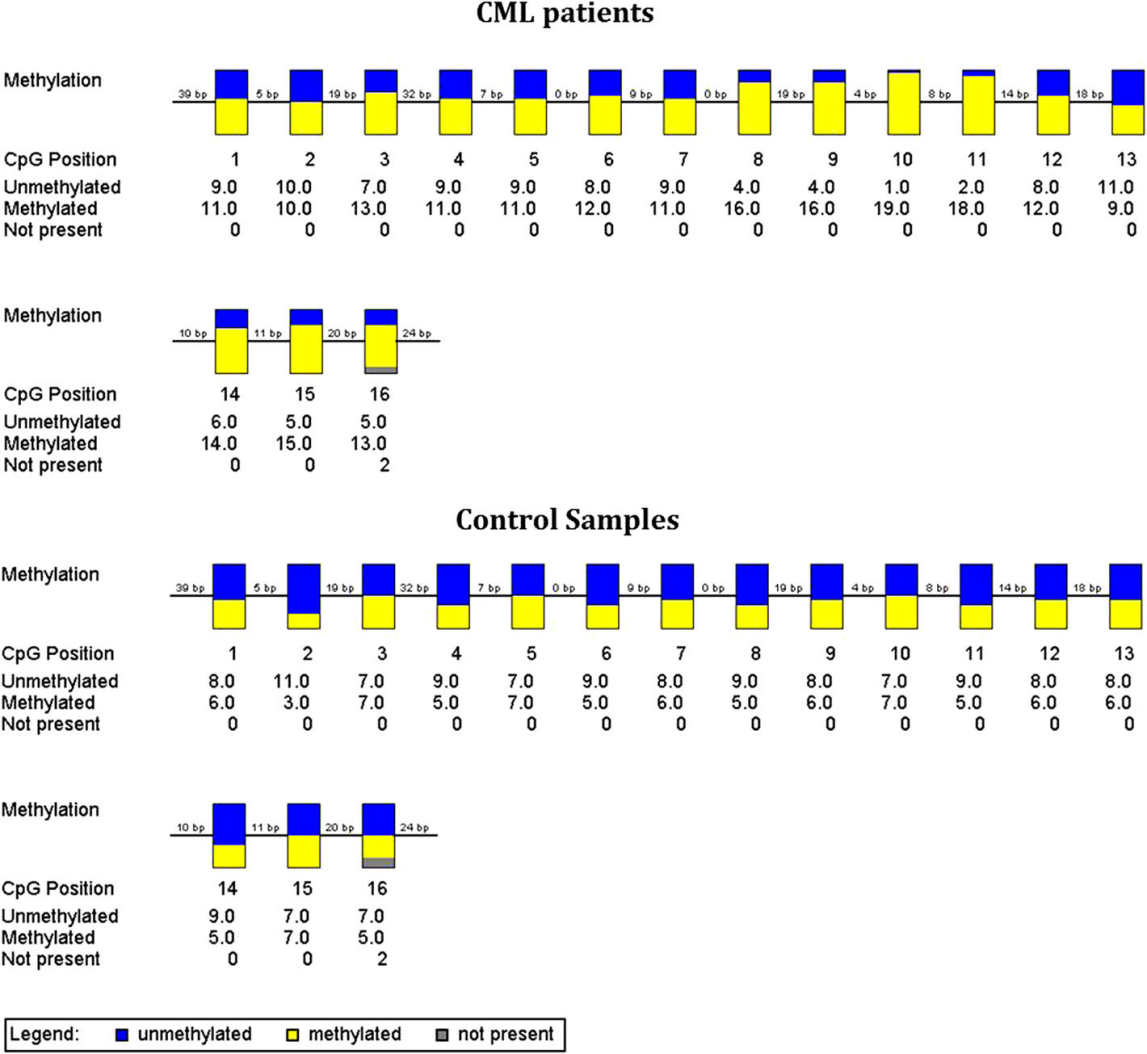
The aggregated representation of methylation data. The Methylation analysis was performed on BSP results for 20 CML patients in different clinical staging and 25 healthy individuals. The relative position of CpG sites is depicted by the gaps between the boxes. The graph was drawn using BiQ Analyzer software v 2.02

According to analysis by BSP and sequencing interpretation, the NOV gene was shown to be significantly hypermethylated in CML patinets compared to healthy individuals. The CpG methylation percentage in the CML patinets in chronic (60.8%), accelerated (75.9%), and blastic (66.7%) phases were significantly greater than those observed in normal subjects (34.8%) (Mann Wittney U-test: *P* = 0.0001). The lollipops plot of methylated and unmethylated CpG sites as well as the aggregated representation of the methylation data are illustrated in Fig. 2 and Fig. 3, respectively.

The methylation of CpG island was observed in 66.4% of CpG sites (ranging from 12.5 to 87.5%; median: 75%) of samples obtained from CML patients and 34.8% (ranging from 12.5 to 75%; median: 25%) of control samples. Collectively, 6 CpG positions showed a significant hypermethylation in CML group (*P*-value of Fisher’s exact test less than 0.05) including the CpG number 1 (*P* = 0.008), 8 (*P* = 0.0003), 9 (*P* = 0.0008), 10 (P = 0.0003), 11 (P = 0.000), 14 (*P* = 0.001).

### The NOV methylation pattern remains intact following imatinib therapy in CML patients

**Fig. 4:**
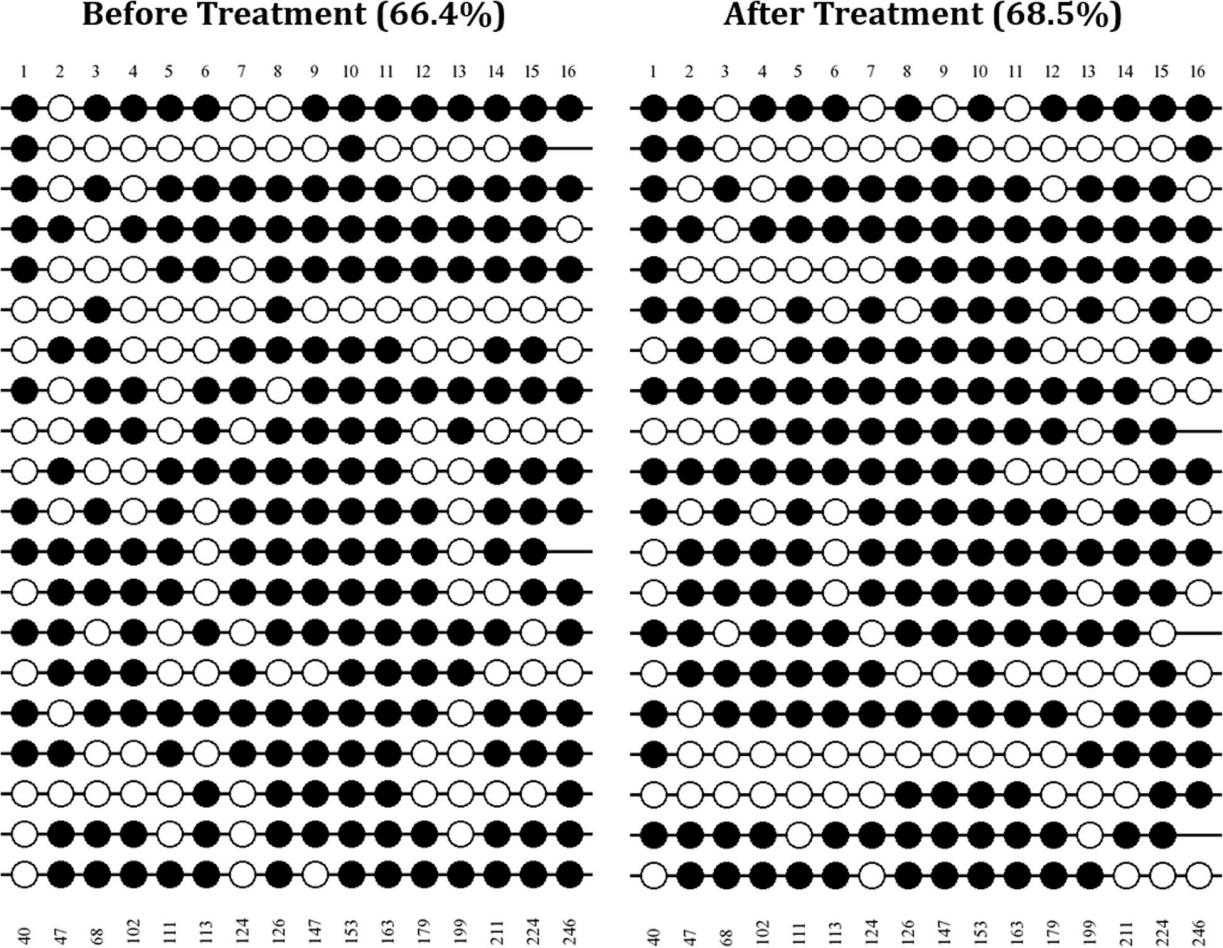
The lollipops representation of the methylation pattern of patients before and after imatinib therapy. The number of CpG position is inserted at the top of each position and the nucleotide position in the target sequence is depicted at the bottom of each CpG site. The Black circles represent the methylated sites while the white circles indicate the unmethylated positions. The diagram was created using QUMA online software

To determine whether the methylation pattern of NOV gene is affected by the suppression of BCR-ABL1 tyrosine kinase activity, we performed the BSP on the patients upon undergoing imatinib therapy. Our BSP analysis showed the mean methylation percentage of 68.5% for the patients undergone imatinib therapy ranging from 25 to 93.8% (mean before treatment = 66.4%) (Fig. 4). Interestingly, our findings demonstrated that while the BCR-ABL1 titers are significantly down-regulated in the patients undergoing imatinib therapy (*P* = 0.011), the NOV methylation patterns remained intact (*P* = 0.978), suggesting that the methylation status of the NOV gene might be an independent risk factor in the CML patients which is not influenced by the BCR-ABL1 downstream events.

### CML patients in different clinical staging do not show variation in NOV methylation

In order to decipher the variation in the methylation status of the NOV gene in the patients with CML in chronic, accelerated, and blastic phases, we performed the Mann-Whitney U-test between the different groups. A comprehensive statistical analysis of the methylation pattern of NOV gene is described in Table 1 for different groups of CML patients. Briefly, it was revealed that while the patients in chronic (*n* = 9), accelerated (*n* = 5), and blastic (*n* = 6) phases are significantly rich in methylated CpG sites compared to the control samples (with *P* value = 0.029, *P* = 0.0008, and *P* = 0.0032, respectively); the statistical data indicated that regardless of the clinical staging, the patients in different CML phases with various BCR-ABL1 titers are not significantly different in terms of NOV methylation levels (Pv of chronic-accelerated analysis = 0.72; chronic-blastic = 0.93; and accelerated-blastic = 0.61), which further validates the assumption that NOV methylation might act as an independent factor during CML pathogenesis.

**Table 1:** The statistical analysis of NOV gene methylation pattern in different phases of CML

CpG position		40	47	68	102	111	113	124	126	147
Me-CpG	Chronic	6/9 66.7%	2/9 22.2%	6/9 66.7%	4/9 44.4%	4/9 44.4%	6/9 66.7%	4/9 44.4%	6/9 66.7%	7/9 77.8%
*P*-value of Fisher’s exact test		0.7041	0.5908	0.1392	1.0000	1.0000	0.1157	0.7041	0.0403	0.0168
CpG position		153	163	179	199	211	224	246		Total
Me-CpG	Chronic	8/9 88.9%	7/9 77.8%	4/9 44.4%	6/9 66.7%	6/9 66.7%	7/9 77.8%	4/8 50.0%		87/143 60.8%
*P*-value of Fisher’s exact test		0.0468	0.0074	0.6870	0.1157	0.0173	0.1251	1.0000		0.0000
CpG position		40	47	68	102	111	113	124	126	147
Me-CpG	Accelerated	3/5 60.0%	4/5 80.0%	3/5 60.0%	3/5 60.0%	4/5 80.0%	2/5 40.0%	4/5 80.0%	5/5 100%	5/5 100%
*P*-value of Fisher’s exact test		1.0000	0.0058	0.6221	0.6424	0.3549	1.0000	0.1377	0.0032	0.0056
CpG position		153	163	179	199	211	224	246		Total
Me-CpG	Accelerated	5/5 100%	5/5 100%	4/5 80.0%	1/5 20.0%	4/5 80.0%	4/5 80.0%	4/4 100%		60/79 75.9%
*P*-value of Fisher’s exact test		0.0447	0.0032	0.1282	1.0000	0.0195	0.3295	0.0932		0.0000
CpG position		40	47	68	102	111	113	124	126	147
Me-CpG	Blastic	2/6 33.3%	4/6 66.7%	4/6 66.7%	4/6 66.7%	3/6 50.0%	4/6 66.7%	3/6 50.0%	5/6 83.3%	4/6 66.7%
*P*-value of Fisher’s exact test		0.3944	0.0138	0.3589	0.3944	1.0000	0.1735	0.6526	0.0132	0.1510
CpG position		153	163	179	199	211	224	246		Total
Me-CpG	Blastic	6/6 100%	6/6 100%	4/6 66.7%	2/6 33.3%	4/6 66.7%	4/6 66.7%	5/6 83.3%		64/96 66.7%
*P*-value of Fisher’s exact test		0.0209	0.0013	0.1735	1.0000	0.0434	0.3944	0.1602		0.0000

### Clinical and laboratory characteristics of CML patients

Of the Nine patients in chronic phase, 8 (88.8%) achieved a Complete Hematologic Response (CHR); while 5 (55.5%) showed a Complete Molecular Response (CMR), 3 (33.3%) achieved Partial Molecular Response (PMR), and 1 (11.1%) with increased BCR-ABL/ABL ratio (NMR) within 6 months of follow up. In the patients with accelerated phase, 3 cases (60%) achieved CHR, and 2 (40%) gained MMR within 11 months of follow up. The patients in blastic phase showed a poorer response, 3 showed the CHR (50%), whereas 2 (33.3%) achieved the MMR, 3 (50%) with PMR, and 1 (16.6%) with NMR. In general, the hematologic and molecular responses showed no significant correlation (*P* = 0.085). Also, no meaningful relationship was found between the patient’s sex, dosage, or duration of imainib consumption (*p* = 1.10, *p* = 0.65, *p* > 0.05). Interestingly, regardless of clinical staging and patient’s response to imatinib treatment, the analysis of NOV promoter showed no significant changes in methylation level among the treated patients.

## Discussion

The presence of the BCR-ABL1 fusion gene as a pathognomonic molecular event in almost all the CML cases has distinguished this malignancy from other myeloproliferative disorders. In spite of significant efforts made to deciphering mechanisms of the disease pathogenesis and elucidation of many BCR-ABL molecular targets, the underlying mechanisms triggering the disease and those responsible for clonal evolution of BCR-ABL-positive clones have not been comprehensively clarified.

Having the integral roles during clonal evolution in leukemia [26–28], the epigenetic mechanisms are considered as ideal candidates to be analyzed for scrutinizing the process of the leukemia initiation and progression. Therefore, we sought to investigate the possible involvement of DNA methylation as the well-known component of the epigenetic machine in the pathogenesis of CML patients in a group of clinical samples confirmed to have CML. Our study revealed that CCN3/NOV, as a key regulator in CML, significantly hypermethylated in the patients compared to normal individuals. By analyzing the clinical samples collected from the different clinical staging of CML patients and also measuring the methylation levels following imatinib therapy, we further validated that the CCN3 remains hypermethylated in the patients following treatment with BCR-ABL1 tyrosine kinase inhibitor, imatinib; as well as in different clinical stages. These findings raise the question that DNA methylation of CCN3 might serve as the molecular event acting upstream of BCR-ABL1 functions. However, supporting this idea requires further efforts analyzing functional consequences using standard experimental platforms.

BCR-ABL1 formation seems to be an early event during CML pathogenesis which also governs numbers of downstream molecular deregulations responsible for cell proliferation, apoptosis resistance, survival, and cell-cycle progression, thus affecting the overall disease progression [29]. Despite this fact, it has been proposed that there might be the epigenetic changes which decide which clones will dominate the stem cell niche and evolve to differentiated functioning cells [30].

Nephroblastoma Overexpressed (NOV) encodes a small secreted protein, which is a member of the CCN family of regulator proteins (CCN3). This family has been reported to regulate the skeletal and cardiovascular development, fibrosis, as well as cancer formation. Based on Gene Ontology (GO) annotations, the association of CCN3 in Notch signaling pathway (KEGG) and cell adhesion has been also described in which it serves a growth factor activity and integrin binding [31].

Gupta et al. reported that the functional integrity of hematopoietic stem and progenitor cells is regulated in large extent by the matricellular protein CCN3 [14]. They showed that the forced expression of recombinant Nov accelerates either the primitive stem or progenitor activity. Their findings supported the idea that CCN3 is an essential regulator of human HSC or progenitor cells.

A previous study introduced the CCN3 as a novel gene to be downregulated in CML cells. It was also supported that this suppression is a direct consequence of BCR-ABL1 kinase activity. This was shown to be a novel downstream event for BCR-ABL activity. Briefly, Lynn McCallum et al. discuss that cellular CCN3 is decreased in the BCR-ABL active FDCB-Mix cells. They also demonstrated that targeting BCR-ABL kinase activity by imatinib or siRNA significantly inhibits the BCR-ABL while increasing the CCN3 levels. Hence, they came to the conclusion that BCR-ABL directly regulates the CCN3 secretion in the CML cells where the levels of cellular CCN3 is reduced. They showed that the level of secretory CCN3 following imatinib therapy not only was not reduced but also increased in extends. Our findings are consistent with this data, in which we have shown that the imatinib therapy did not affect the levels of CCN3 gene hypermethylation, a possible mechanism for the gene suppression. In case of hierarchical events, our findings might be in contrast to those obtained by the McCallum’s study. However, the gene expression can be regulated at various molecular levels. Interestingly, they also transfected the CCN3 into the BCR-ABL+ cells, and observed that the stem cell proliferation and clonogenic potential were significantly suppressed. While the CCN3 regulation was considered as a downstream event of BCR-ABL in their study, we suggest that there might be mechanisms acting beyond the BCR-ABL function to prone the BCR-ABL+ clones dominating the stem cell niche and thereby progress to CML. We observed that even the patients with sub-optimal molecular responses (less than 3-log decrease in BCR-ABL1 transcripts compared to the baseline standard levels) or no molecular response showed the NOV hypermethylation, and no significant changes was observed in the level of NOV methylation in various clinical staging with the treatment response. However, the McCallum’s results showed that the CCN3 expression was up-regulated following BCR-ABL inhibition. This might be interpreted as a non-methylation mechanism for CCN3 reactivation in this context. In addition, although the McCallum’s study argues that the CCN3 secretion is controlled by direct activity of BCR-ABL, they verify the downregulated levels of cellular CCN3 in CML cells. Therefore, it can be assumed that the secretory and cellular CCN3 might be regulated through different mechanisms at distinct cellular levels [20].

A distinct study reported that despite the undetectable CCN3 levels in CML stem cells, the progenitor cells respond to recombinant CCN3. In this study McCallum et al. showed that abrogation of CCN3 accelerates the leukemogenesis mediated by BCR-ABL, highlighting the important growth regulatory function of CCN3 in hematopoiesis [21].

In another attempt to clarify the mechanism of CCN3 downregulation in CML, Suresh et al. assessed the role of MicroRNAs during BCR-ABL mediated signaling pathway. They hypothesized that BCR-ABL regulates a range of oncogenic and tumor suppressor miRNAs and thereby favors the malignant behaviors. They reported that miR-130a can significantly suppress both the levels of CCN3 mRNA and protein in HL60 cells. It was concluded that miRNA profile which is deregulated as a result of BCR-ABL activities can be one of the mechanisms for down-regulation of CCN3 through which the leukemic cells evade the negative growth regulation [22].

In a distinct study, McCallum et al. assessed more deeply the role of CCN3 during the leukemogenic process. They validated that CCN3-expressing K562 cells significantly reduce the cell growth, colony forming capacity, and mitogenic signaling while compared with the cells transfected with empty vectors. They also indicated that the CCN3 overexpressed cells accelerated the apoptosis through 3-fold increase in annexin V and affirmed that this apoptosis induction remains even after imatinib treatment. These findings, combined with other results showing that the transfected cells display more robust adhesive potentials highlighted the role of CCN3 as a growth regulator which also sensitizes the CML cells to imatinib-induced apoptosis. Their findings supported the idea that CCN3 can open novel doors for alternate anti-leukemia therapeutics [32].

Although recent studies have shown distinct mechanisms for CCN downregulation in CML and that this inhibition occurs as a direct BCR-ABL kinase activity, the epigenetic mechanisms for CCN3 suppression had been poorly understood. In the current study, we further demonstrated that deregulation of this gene might be at least in part due to hypermethylation of the promoter, which can act as an early event during the leukemogenesis. Having clonogenic activities and being involved in several properties of hematopoietic stem cells, we assume the CCN3 hypermethylation as a candidate initiator of CML, which can provide the clonal domination of BCR-ABL+ cells. However, further studies are required to clarify the exact influence of NOV DNA hypermethylation by analyzing a comprehensive set of signaling pathways involved in the disease.

## Conclusions

Collectively, our findings validated a hypermethylated pattern for NOV/CCN3 gene in CML patients, which is not influenced by the imatinib therapy, suggesting this as a BCR-ABL independent molecular event, which raises the question that whether the CCN3 hypermethylation serves as an early trigger for CML formation. Based on our findings and previous results from other studies regarding the role of CCN3 in stem cell function and clonal evolution, this molecular target can provide alternative therapeutic strategies for CML management.

## Abbreviations

BSP: Bisulfite PCR
CBC: Complete blood count
CCN3: Cellular Communication Network Factor 3
CML: Chronic Myelogenous Leukemia
EDTA: Ethylenediaminetetraacetic acid
Go: Gene Ontology
HSC: Hematopoietic Stem Cells
NOV: Nephroblastoma Overexpressed
qPCR: Quantitative Polymerase Chain Reaction
